# Papulosquamous Disorders and Pregnancy

**DOI:** 10.7759/cureus.18762

**Published:** 2021-10-13

**Authors:** Amal O AlBalbeesi, Tala A Qadoumi

**Affiliations:** 1 Dermatology, King Saud University, Riyadh, SAU

**Keywords:** skin disorders, pregnancy outcome, lichenoid disorders, papulosquamous disorders, gestation, pregnancy

## Abstract

Pregnancy can influence the course of a number of dermatologic disorders. Interestingly, these disorders can also influence pregnancy outcomes negatively, due to a variety of underlying pathogenic mechanisms. These outcomes may range from low fetal birth weight, preterm birth, and miscarriages to perineal lacerations complicating deliveries. Our review highlights the impact that papulosquamous disorders may have on pregnancy and their course throughout pregnancy. We chose papulosquamous disorders due to their relatively high prevalence worldwide compared to other dermatologic disorders. This review also sheds light on any gaps in the literature relevant to this topic that should be addressed.

## Introduction and background

Pregnancy is a time of immunologic, metabolic and hormonal changes, which can influence the course of a number of dermatologic conditions, many of which have an underlying immunologic nature. Skin manifestations during pregnancy can include physiologic changes such as hyperpigmentation, vascular skin lesions, and striae distansea [1,2]. Other cutaneous manifestations range from pregnancy-specific dermatoses such as polymorphic eruption of pregnancy, atopic eczema of pregnancy, and pemphigoid gestationis [3] to primary dermatoses unrelated to pregnancy including psoriasis and pityriasis rosea. Our review focuses on these primary dermatoses, specifically papulosquamous diseases. Pregnancy can influence the prognosis of these conditions, with some worsening throughout pregnancy or the post-partum period, others improving or stabilizing [4]. Furthermore, certain dermatoses were found to possibly impact pregnancy outcomes resulting in stillbirths, low birth weight, or prematurity amongst other complications, emphasizing the need for closer follow-ups and prompt management.

## Review

Methodology

The studies were located on PubMed using the keywords “pregnancy”, “gestation”, “pregnancy outcome”, and/or “named papulosquamous disorders”. Disorders named in the search included psoriasis, pustular psoriasis (and impetigo herpetiformis), pityriasis lichenoides, pityriasis rosea, pityriasis rubra pilaris, parapsoriasis, lichen planus, and lichen sclerosus et atrophicus. Relevant studies published from January 1, 2000 to December 31, 2021 were included in this review. Titles and abstracts were obtained and reviewed and studies that mentioned the impact of the specific disorder on pregnancy outcome and its course throughout pregnancy were included. Any other studies, including studies published outside the above-mentioned time frame, were excluded from this review. Studies regarding management of these disorders during pregnancy were also excluded. Duplicate results were excluded. Reference lists of the included studies were also extensively searched for any other relevant studies. The search resulted in 450 articles in total, 38 of which were included in this review.

Psoriasis

Psoriasis is a chronic inflammatory disorder that affects 2% of the world’s population [5]. Psoriasis is multifactorial and immune mediated, where CD4+ T cells, either Th1 cells or Th17 cells, play an important role [6]. Around half of patients with psoriasis are women. Three-quarters of these patients are diagnosed at the age of 40 or younger, making psoriasis prevalent during reproductive years [7]. The course of psoriasis can be difficult to predict throughout pregnancy. Around 50% of women with psoriasis experience clinical remission during pregnancy, others (18% to 21%) show no change while some (23% to 26%) can show worsening [8,9]. Moreover, 65% of patients experience worsening during the post-partum period [9]. The Th2-mediated immune response is heightened during pregnancy which downregulates the Th1-mediated immune response responsible for psoriasis [10]. This explains why the majority of pregnant women with psoriasis improve during pregnancy.

Psoriasis and other inflammatory conditions such as rheumatoid arthritis, systemic lupus erythematosus, and inflammatory bowel disease are known to be associated with adverse pregnancy outcomes, such as low birth weight, preterm birth, and abortions [11]. Lima et al. found in their retrospective cohort that psoriasis was associated with a 1.89 fold increased risk of a poor outcome composite which they defined as a low birth weight (less than 2,500 g) and preterm birth (less than 37 weeks) [12]. Another retrospective cohort carried out by Cohen-Barak and colleagues included 68 deliveries to 35 women with moderate to severe plaque psoriasis and compared them to 237 deliveries in 236 matched women with no psoriasis. Pregnancies of women with psoriasis were found to be associated with spontaneous and induced abortions, pregnancy-induced hypertensive diseases, premature rupture of membranes, large-for-gestational-age newborns, and macrosomia [13]. A population-based cohort study done in Denmark and Sweden included 8,097 births in 6,103 women with psoriasis. In this study, psoriasis was associated with an increased risk of gestational diabetes, gestational hypertension, pre-eclampsia, elective and emergency caesarean delivery. Severe psoriasis was associated with an increased risk of low birth weight and preterm birth, as well as an increased risk for other poor outcomes mentioned above. Patients were classified as having severe psoriasis if they were receiving systemic treatments and have dispensed at least once, one of the following systemic agents: methotrexate, cyclosporine, systemic anti-psoriatic treatments and/or biologic agents such as tumor necrosis factor inhibitors, ustekinumab, leflunomide, efalizumab, abatacept before or during pregnancy [14]. Ben-David et al. evaluated 145 deliveries in women with psoriasis in a case-control study compared to 860 deliveries in non-psoriatic women. Psoriasis was found to be an independent risk factor for caesarean delivery. Psoriasis was also found to be significantly associated with chronic hypertension and recurrent abortions [15].

Another entity of psoriasis is pustular psoriasis. Pustular psoriasis of pregnancy (PPP), also known as impetigo herpetiformis, has been classically viewed as a specific dermatoses of pregnancy [16]. Recent data suggests that it may be a variant of generalized pustular psoriasis with significant fetal and maternal morbidity [16], making it worth mentioning in this review. There is however an ongoing debate on whether PPP is a pregnancy-specific dermatoses or a subtype of psoriasis. A number of causes have been postulated as part of the underlying etiology of PPP, including mutations in interleukin 36 receptor antagonist (IL36RN), which encodes the IL36 receptor antagonist [17]. Another possible cause is hypocalcemia [17], which may be triggered by pregnancy, owing to increased nutritional demands. PPP typically appears during the third trimester of pregnancy as grouped pustules over erythematous patches and plaques, that begin in intertriginous areas and progress centrifugally to involve the face, palms and soles [18]. Lesions are associated with systemic symptoms such as fever, diarrhea, tetany and delirium [18]. The lesions may then progress to erythroderma, resulting in fluid and electrolyte imbalances, secondary infections, sepsis and maternal morbidity [19]. PPP usually resolves rapidly during the post-partum period, however there is a case report documenting a post-partum flare [20].

PPP also has a significant effect on the fetus, with a risk of placental insufficiency, premature rupture of membranes, preterm labor, intrauterine growth restriction and stillbirth, prompting close monitoring, early recognition and intervention [17,21]. Fetal prognosis is often worse than maternal prognosis, with a higher risk for still-birth as compared to maternal death [21]. A case reported by Kondo et al. highlighted the difference in pregnancy outcomes in two successive pregnancies. The first pregnancy resulted in stillbirth while the second resulted in a healthy baby, due to the institution of early systemic treatment (systemic steroids and antibiotics) and recommendation of delivery resolution [21]. Two separate case reports from China detailed two different PPP cases that were both managed with termination of pregnancy, due to lack of response to conventional systemic therapies, such as systemic steroids and antibiotics [22,23]. Typically, PPP will recur in subsequent pregnancies, with an earlier onset and a more severe presentation [17,24]. 

Pityriasis rosea

Pityriasis rosea (PR) is a common self-limited papulosquamous disorder that is typically associated with human herpes virus (HHV) 6/7 reactivation. It usually presents in individuals during the second or third decade of life as a single erythematous patch initially followed by an eruption of smaller similar patches. The altered immune response in pregnancy explains the increased risk for reactivation of the human herpes viruses as well as why pityriasis rosea is found more commonly in pregnant women (18%) compared to the general population (6%) [25]. However, PR is not without its complications in pregnant women and has been found to negatively impact pregnancy outcome. Drago and colleagues found in a case series that included 38 women diagnosed with PR during their pregnancies, that 62% of these women, who were diagnosed with PR before 15 weeks of gestation, aborted. Nine of them experienced a premature delivery and neonatal hypotonia, weak motility, and hyporeactivity was found in six cases [26]. Drago et al. carried out another study that evaluated risk factors associated with a negative pregnancy outcome in patients diagnosed with PR during pregnancy. Early onset of PR, occurring prior to 15 weeks of gestation, and presence of enanthem were significantly associated with pregnancy complications and considered to be major risk factors. Other risk factors (considered to be minor risk factors) for pregnancy complications included a large body surface area affected (more than 50%), presence of constitutional symptoms, and the viral load of HHV-6 measured in copies/ml [27]. A case report published in 2016 by Loh and Cohen detailed the occurrence of a possibly coincidental isolated sagittal craniosynostosis in a fetus whose mother acquired pityriasis rosea during the first trimester of pregnancy, with no other pregnancy complications [28]. The most recent study regarding pityriasis rosea’s implication in adverse pregnancy outcomes was a retrospective multicenter study carried out by Stashhower et al. This study included 33 patients and 24% of them had birth complications, which were considered to be minor with no history of abortions, miscarriages or fetal deaths. They also found that the women who had birth complications were diagnosed, on average, with PR earlier as compared to those who did not have complications, however this finding was not found to be significant [29].

Pityriasis lichenoides et varioliformis acuta (PLEVA) and other papulosquamous disorders

Pityriasis lichenoides can present as a wide spectrum of manifestations with multiple variants, involving both the skin and mucous membranes. One of its variants is pityriasis lichenoides et varioliformis acuta, or PLEVA, which presents as an acute to subacute eruption of erythematous cutaneous papules that can evolve into polymorphic lesions, with varioliform scarring upon resolution [30]. Pityriasis lichenoides is more commonly seen in children and young adults [31]. A few case reports have been published in literature of PLEVA occurring in pregnant women. Lesions affecting the mucous membranes of the vagina and cervix have been implicated with premature labor and/or rupture of membranes [32,33].

There are not many studies evaluating the impact of parapsoriasis on pregnancy [34]. One study evaluated the impact that mycosis fungoides and parapsoriasis en plaque may have had on pregnancy in 12 pregnant women, nine of whom had mycosis fungoides while three had parapsoriasis. They found no adverse effect of these conditions on pregnancy outcome and no effect of pregnancy on these disorders [34]. Similarly, there are no papers, to our knowledge, studying the impact that pityriasis lichenoides chronica or pityriasis rubra pilaris may have on pregnancy.

Lichen sclerosus et atrophicus and other lichenoid disorders

Lichen sclerosus (LS) is a chronic inflammatory disease, mainly affecting the anogenital region. The prevalence of LS increases with age, but it may affect any age group, with a predominance in women as compared to men [35]. Lichen sclerosus has significant long-term consequences including alteration of vulvar architecture, possibly impacting the mode of delivery. LS is also associated with an increased risk of vulvar carcinoma. However, there is little literature looking into the effect that LS may have on pregnancy and its course throughout pregnancy.

A recent study carried out in 2019 by Trokoudes and Lewis assessed 36 pregnancies in 22 patients with lichen sclerosus. All patients were managed with clobetasol proprionate ointment and none experienced any worsening during pregnancy, except for one patient who had a flare post partum. Thirty-three deliveries were carried out vaginally, while three deliveries were done via a caesarean section, due to failure to progress. There were 15 cases of obstetric tears, one of which was a third-degree tear in a patient whose LS was not treated during pregnancy. That same patient experienced no complications during her second pregnancy when her LS was appropriately managed and was inactive at the time of her pregnancy [36]. Another retrospective review included 33 patients diagnosed with vulvar lichen sclerosus. Eight of these patients became pregnant after their diagnosis. Of the eight deliveries, three had vaginal deliveries, two of which developed second-degree lacerations. Four of the deliveries were cesarean deliveries. It is unclear whether vulvar lichen sclerosus aggravated the risk of obstetric tears. Furthermore, 63% of the patients were symptomatic during pregnancy, whilst 25% had exacerbations. Three of these patients were noncompliant to the medications and two were not advised to apply any treatments, due to concerns of side effects of these medications during pregnancy [37].

Similarly, Nguyen et al. found in their retrospective review of 33 deliveries to 29 women with biopsy-confirmed lichen sclerosus, that the majority (82%) delivered vaginally, two delivered instrumentally and four had caesarean sections. Only one of the caesarean sections was due to extensive scarring in the vulvar region as a result of non-compliance to medication [38]. 

To our knowledge, there are no studies assessing the course of lichen planus during pregnancy or its impact on pregnancy outcome. This provides fertile ground for further studies into this topic as well as other dermatoses during pregnancy. 

## Conclusions

Knowledge regarding how different dermatologic disorders affect pregnancy is essential as it calls for a closer observation of the fetus and pregnancy, as a whole. It also allows for the prediction of and preparation for any issues that may arise throughout pregnancy and at the time of delivery. This, in turn, facilitates more effective management. On the other hand, knowledge of the course that these disorders normally take during pregnancies, allows physicians to remain on the lookout for possible complications and promptly make the appropriate adjustments in their treatment plan to address any possible flares or remissions. Despite the aforementioned reasons, literature in this topic is still lacking. For instance, compared to the prevalence of psoriasis worldwide especially in women of child-bearing age, studies evaluating the effect psoriasis has on pregnancy outcome are scarce. These studies also highlight the importance of disease control prior to conception and throughout pregnancy.This review allowed us to identify the gaps in literature, which provides ample opportunities for essential future research.
